# Epidemiologic and economic impact of introducing a National Immunization Program for herpes zoster vaccination on the prevention of herpes zoster and postherpetic neuralgia in South Korea, an ageing society

**DOI:** 10.1017/S0950268826101502

**Published:** 2026-04-30

**Authors:** Gahee Kim, Jaehee Jung, Dain Lee, Hee-Do Yang, Hye-Young Kang

**Affiliations:** 1 Department of Pharmaceutical Medicine and Regulatory Sciences, College of Medicine and College of Pharmacy, Yonsei University, Republic of Korea; 2 Yonsei Institute of Pharmaceutical Science, College of Pharmacy, Yonsei University, Republic of Korea; 3 Graduate Program of Industrial Pharmaceutical Science, College of Pharmacy, Yonsei University, Republic of Korea

**Keywords:** cost savings, herpes zoster, live-attenuated vaccine, postherpetic neuralgia, recombinant zoster vaccine

## Abstract

Herpes zoster (HZ) and its complication, postherpetic neuralgia (PHN), disproportionately affect older adults and impose a considerable disease and economic burden. Although the Korea Disease Control and Prevention Agency recommends vaccination for adults aged ≥50 years, uptake remains limited. This study assessed the epidemiologic and economic impact of expanding vaccine coverage through inclusion of the live-attenuated zoster vaccine (ZVL) and the recombinant zoster vaccine (RZV) in the National Immunization Program (NIP). Using the incidence data from the Health Insurance Review and Assessment Service Open Statistics, along with vaccine effectiveness, coverage rates, and treatment cost estimates adjusted to 2025 values, we projected cases averted and associated cost savings. At the current 10% coverage, ZVL and RZV were estimated to prevent 36269 and 56681 HZ cases and 15988 and 20712 PHN cases, yielding societal cost savings of USD 78.47 million and USD 107.14 million, respectively. Expanding NIP coverage to 70% amplified benefits approximately sevenfold, yielding cost savings of USD 549.34 million (ZVL) and USD 749.98 million (RZV). These results demonstrate the substantial value of zoster vaccination and underscore the need for policy measures to improve vaccine coverage among older adults in South Korea.

## Introduction

Herpes zoster (HZ), commonly known as shingles, is caused by the reactivation of latent varicella-zoster virus and typically affects older adults and immunocompromised individuals [1, 2]. It is characterized by a painful rash and blisters, usually localized to one side of the body or face [1]. In South Korea, the incidence of HZ has substantially increased over time, rising from 2.67 to 9.80 per 1000 person-years between 2003 and 2015 [3]. According to the Health Insurance Review and Assessment Service (HIRA) Open Statistics, the number of patients aged 80 and older treated for HZ increased by approximately 60.3% from 43306 in 2015 to 69423 in 2023 [4]. This substantial rise in the number of patients, driven by a rapidly ageing population, signifies a growing public health burden among older adults in South Korea. Although the reasons for varicella zoster virus reactivation are not fully understood, the risk of developing HZ is particularly high among older adults and individuals with chronic conditions commonly seen in this age group, such as diabetes, chronic obstructive pulmonary disease, and chronic kidney disease [5]. This upward trend in South Korea aligns with a global pattern observed in ageing populations [6].

Postherpetic neuralgia (PHN), a chronic neuropathic pain condition following HZ, represents a major complication that can persist for months or even years after the initial outbreak of HZ [7]. PHN is associated with substantial physical and psychological burden, including sleep disturbance, reduced mobility, and diminished quality of life [8]. An estimated 10%–18% of patients with HZ develop PHN, with increasing risk observed in older age groups [9]. The analysis of 2021 HIRA claims data showed that age-specific PHN conversion rates in South Korea ranged from 7.9% among individuals aged 50–54 to 17.7% in those aged 75 and older [10]. Across multiple countries, the medical cost burden for HZ cases accompanied by PHN has been shown to be approximately two to five times higher than for uncomplicated HZ alone [11–13].

Currently, two types of HZ vaccines are licensed in South Korea: live-attenuated vaccines (ZVL), including Zostavax® (MSD, introduced in 2013) and Sky Zoster® (SK Bioscience, introduced in 2017), and a recombinant zoster vaccine (RZV), including Shingrix® (GSK, introduced in 2022) [14]. All three vaccines are approved for use in individuals aged 50 years and older [14]. In response to the growing burden of HZ, the Korea Disease Control and Prevention Agency (KDCA) and the Korean Society of Infectious Diseases (KSID) recommended both the ZVL and RZV for adults aged 50 years in its 2023 adult immunization guidelines [15, 16].

Despite proven effectiveness and official recommendations, HZ vaccines are not included in the National Immunization Program (NIP) in South Korea, and coverage remains low at approximately 10% [10, 17, 18]. One of the primary barriers to vaccine uptake is its high out-of-pocket cost, cited as the main reason for non-vaccination in a multi-centre survey [18]. These findings underscore the need to reduce financial barriers to improve uptake, especially among high-risk groups. In recent years, integrating HZ vaccination into the NIP has gained attention as a public health priority in South Korea [19]. This aligns with evidence that the risk of HZ and its most severe complication, PHN, rises markedly after the age of 50 [9, 10], consistent with the current vaccine recommendations.

This study aims to quantify the potential epidemiologic and economic benefits of expanding ZVL and RZV vaccination for Korean adults aged 50 and older through inclusion in the NIP. We estimated the number of preventable HZ and PHN cases, along with their associated costs, under a scenario in which vaccination coverage reaches levels observed in countries with publicly funded HZ vaccination. We expect that our findings offer timely and actionable evidence for policymakers seeking to optimize adult immunization strategies in response to a rapidly ageing population and rising healthcare expenditures.

## Methods

### Overview of the methods

This study estimated the number of preventable cases of HZ and PHN and the corresponding cost savings for a single year, following the introduction of the NIP. The number of preventable cases of HZ and PHN was estimated by combining the population size of adults aged 50 and above, the incidence rates of HZ and PHN among those unvaccinated, vaccine coverage rates, and vaccine effectiveness (VE). To assess the reduction in National Health Insurance (NHI) spending due to these preventable cases, the estimated number of avoided HZ and PHN cases was multiplied by the corresponding NHI-covered medical cost per case.

Furthermore, we analysed the potential healthcare cost savings across different market share scenarios for ZVL and RZV vaccines. All costs are reported in 2025 US dollars (USD), applying an exchange rate of 1 USD to 1300 Korean won (KRW). The input data were sourced from published studies, national health statistics, and other secondary datasets. To enhance the transparency and reproducibility of our model, all input parameters – including base-case values, ranges used in sensitivity analyses, and data sources – are summarized in Supplementary Appendix Table C1. Detailed explanations of each parameter’s source and application are provided in the following sections.

It is important to note that the primary objective of this study was to quantify the epidemiological impact (prevented cases) and the cost savings attributable to averted HZ and PHN episodes, rather than to conduct a comprehensive budget impact analysis including vaccination expenses. In South Korea, treatment costs for HZ and PHN are primarily covered under the NHI system, administered by the National Health Insurance Service (NHIS), with additional patient cost-sharing and out-of-pocket payments for non-covered services. In contrast, if HZ vaccination were to be introduced into the NIP, vaccine procurement and programme costs would be financed through government immunization budgets and implemented under the oversight of the KDCA. Currently, HZ vaccines are not part of the NIP and are largely paid out-of-pocket by patients. Under an NIP setting, future government procurement prices are uncertain. Therefore, vaccination expenses were not included in the present analysis.

### Estimating incidence rates of HZ and PHN in the unvaccinated population

The overall incidence rates of HZ and PHN for 2025 were assumed to be equivalent to those calculated from the 2023 HIRA Open Statistics, which is the most recent data available in South Korea, using the International Classification of Diseases Codes 10^th^ version (ICD-10 codes) B02 and G53.0, respectively [4]. The incidence rates among unvaccinated individuals were then estimated using the overall incidence, vaccination coverage, and VE, following the methodology outlined in Supplementary Appendix A. Separate VE values for ZVL and RZV were applied in the estimation to reflect the distinct effectiveness of each vaccine.

### Assumption of vaccine uptake rates under the NIP

The current vaccination rate was assumed to be 10%, based on previous Korean studies [10, 17, 18]. Following the introduction of the NIP, the vaccination rate was assumed to increase to 70%. This assumption was based on two domestic studies: one projected that a 70% uptake among adults aged ≥65 years would be a realistic target under an NIP-supported setting [17] and another also adopted a 70% rate, reflecting expert consensus that HZ vaccine uptake would be slightly lower than the 80% coverage historically observed for the elderly influenza NIP [10].

### Systematic review of VE against HZ and PHN

To identify robust and contemporary VE values for ZVL and RZV, we conducted a systematic review of systematic reviews and meta-analyses following PRISMA guidelines. We searched PubMed, EMBASE, and the Cochrane Library for systematic reviews and meta-analyses reporting quantitative VE or efficacy estimates published between January 2020 and February 2026. The search strategy combined terms related to herpes zoster, varicella-zoster virus, available vaccines (ZVL, RZV), vaccine efficacy/effectiveness, and methodological filters identifying systematic reviews and meta-analyses. Detailed search strategies, study selection, and methodological quality assessment using AMSTAR-2 are provided in Supplementary Appendix B.

From 176 records identified (150 unique records after de-duplication), titles/abstracts were screened, and three comprehensive systematic reviews/meta-analyses were included [20–22]. For base-case model inputs, we prioritized real-world effectiveness estimates derived from observational studies. Although efficacy estimates from pivotal RCTs provide high internal validity, they are generated under controlled conditions and may not fully reflect vaccine performance in routine clinical practice (e.g., broader populations, adherence to two-dose series of RZV, and real-world healthcare-seeking behaviour).

Accordingly, we selected Mbinta et al. as the primary base-case data source. Mbinta et al. synthesized post-licensure observational evidence for ZVL (22 studies involving 9536086 participants) and included post hoc real-world effectiveness evidence for RZV, such as a large cohort study of 15589546 adults. It also reports age-stratified effectiveness for ZVL against HZ in 10-year bands (50–59, 60–69, 70–79, and ≥ 80 years). Because VE estimates reported in explicit 5-year age intervals were not available in the robust literature, the 10-year estimates were applied uniformly to the corresponding 5-year subgroups in our model (e.g., the 50–59 years estimate was applied to both 50–54 and 55–59 years) [20].

Based on Mbinta et al., the real-world VE of a complete two-dose RZV regimen against HZ was 79.2%. For PHN, Mbinta et al. reported a two-dose VE estimate of 76.0% based on post-licensure observational evidence via forward citation searching. For ZVL, age-stratified effectiveness against HZ declined with age (60.0% for 50–59 years, 50.9% for 60–69, 46.6% for 70–79, and 43.9% for ≥80). The pooled ZVL effectiveness against PHN was estimated at 59.7% [20]. All VE inputs and their uncertainty ranges used in sensitivity analyses are summarized in Supplementary Appendix Table C1.

### Estimating treatment costs for HZ and PHN

The base case cost analysis was conducted from a healthcare system perspective, considering only NHI-covered and non-covered direct medical costs. Per-case costs for treating HZ and PHN were obtained from a cost-effectiveness study by Cheong et al. [10], which utilized 2021 Korean National Health Insurance Service (NHIS) claims data to estimate individual treatment costs. These estimates reflect the healthcare system perspective. NHI-covered costs – comprising outpatient treatment costs, prescribed medication costs, and inpatient hospitalization costs – were sourced directly from the 2021 NHIS claims data. Non-covered costs were estimated by applying service-specific patient cost-sharing ratios, as reported in the 2021 NHI Patient Medical Expenses Survey, to the NHI-covered costs [10]. Total direct medical costs were calculated as the sum of the NHI-covered and non-covered expenditures. To align with the economic context of our study period, we adjusted these costs to 2025 values using NHIS Medical Cost Conversion Index [23]. An additional analysis was conducted from a societal perspective, which expanded the cost components to include transportation expenses for healthcare visits, caregiver costs, and productivity losses. To improve transparency regarding cost drivers, we report the key healthcare resource utilization parameters underlying the per-case cost estimates (e.g., outpatient visits and hospitalization days per case) for both HZ and PHN by 5-year age groups (Supplementary Appendix Table C1). These inputs were taken from a published Korean study based on National Health Insurance claims data [10]. The claims-based utilization measures serve as proxies for the intensity and duration of care, which are typically greater for PHN than for uncomplicated HZ.

### Sensitivity analysis

To assess parameter uncertainty, we conducted one-way sensitivity analyses. VE was a key parameter due to its direct influence on the estimated number of preventable HZ and PHN cases. For both ZVL and RZV, VE values were varied using the lower and upper bounds of the 95% confidence intervals reported by Mbinta et al. [20].

To reflect variability in plausible vaccination uptake under the NIP, an additional sensitivity analysis was performed using a range of vaccination coverage assumptions (50%–90%). This range was chosen based on international data from countries with publicly funded HZ vaccine programmes: England (58%–63%), Australia (60%–65%), and Canada (initially assumed at 80%) [24–27].

Furthermore, because case identification based on administrative ICD-10 codes (B02 for HZ and G53.0 for PHN) may be subject to misclassification – particularly for PHN – we conducted additional one-way sensitivity analyses varying the age-specific baseline incidence rates of HZ and PHN by ±20%. This pragmatic range was used to reflect uncertainty in claims-based case definitions and is consistent with the parameter ranges used in a recent Korean economic evaluation of HZ vaccines [10].

Additionally, for RZV, the vaccination coverage in our base-case analysis represents completion of the two-dose series (i.e., full-series coverage), as the effectiveness inputs were derived from the complete regimen. However, because RZV requires a two-dose series, real-world completion may be suboptimal. To address this and reflect uncertainty in adherence – which has been highlighted as an important driver of the two-dose RZV regimen’s value in prior economic evaluations [28, 29] – we conducted an additional scenario analysis. Specifically, we fixed the initial dose-1 uptake at 70% under the NIP assumption and varied the second-dose completion rate among these recipients from 50% to 100%. This approach resulted in an effective full-series coverage ranging from 35% to 70% (Supplementary Appendix Table C5). Because our base-case VE inputs were parameterized from post-licensure observational evidence on the complete two-dose series, and comparable real-world effectiveness estimates for a single dose were limited for model parameterization, we conservatively assumed zero protection for incomplete regimens in this scenario analysis.

## Results

### Estimated impact of vaccination by coverage scenario

At the current 10% vaccination coverage, ZVL is estimated to prevent approximately 36269 HZ cases in the analysis year (2025), resulting in cost savings of USD 11.31 million from the healthcare system perspective and USD 19.42 million from the societal perspective (Table 1). It is also projected to avert 15988 PHN cases, leading to savings of USD 37.30 million and USD 59.05 million from the healthcare system and societal perspectives, respectively (Table 2). If vaccination coverage increases to 70% with the introduction of the NIP, the number of preventable HZ cases is expected to rise to 253880, with corresponding healthcare system cost savings of USD 79.14 million and USD 135.96 million from a societal perspective (Table 1).

**Table 1.** Single-year estimated number of HZ cases and associated costs prevented by ZVL in adults aged 50+ years in South Korea

Age, years	No. population^a^	No. individuals with HZ^b^	Incidence rate of HZ, %	Vaccination rate, %	Incidence rate of HZ among the unvaccinated, %	VE of ZVL against HZ^c^, %	Expected number of HZ cases prevented by ZVL	Expected HCS cost savings from HZ cases prevented by ZVL, US dollars	Expected societal cost savings from HZ cases prevented by ZVL, US dollars
	A	B	C = B/A × 100	D	E = C/{(1-F) × D +(1-D)}	F	G = A × D × E × F		
50–54	4525612	99422	2.20	10.00 [70.00]	2.34	60.00	6346 [44423]	1505951 [10541657]	3208493 [22459449]
55–59	4070751	107994	2.65	10.00 [70.00]	2.82	60.00	6893 [48253]	1805332 [12637327]	3626942 [25388594]
60–64	4258205	130796	3.07	10.00 [70.00]	3.24	50.90	7015 [49102]	2063738 [14446169]	3505291 [24537040]
65–69	3274183	114014	3.48	10.00 [70.00]	3.67	50.90	6115 [42802]	1882731 [13179118]	3014039 [21098276]
70–74	2234677	79558	3.56	10.00 [70.00]	3.73	46.60	3889 [27220]	1307291 [9151040]	1981213 [13868490]
75–79	1637098	57787	3.53	10.00 [70.00]	3.70	46.60	2824 [19771]	1070373 [7492609]	1519883 [10639179]
80+	2289858	69412	3.03	10.00 [70.00]	3.17	43.90	3187 [22310]	1669755 [11688284]	2566690 [17966827]
Total	22290384	658983					36269 [253880]	11305172 [79136205]	19422551 [135957855]

a Data source: Korea Statistical Information Service (KOSIS), 2023.

b Data source: Health Insurance Review and Assessment Services (HIRA) Open Statistics in 2023. Diagnosis of HZ was defined according to the International Classification of Diseases Code, 10th version (ICD-10 code) of B02.

c Data source: Mbinta JF, et al. (2022) [20].

HCS, healthcare system; HZ, herpes zoster; VE, vaccine effectiveness; ZVL, live-attenuated vaccine. All cost estimates are reported in US dollars (1 USD = 1300 Korean won). Values in [ ] represent estimates based on the assumed vaccination rate after reaching the target level following the introduction of NIP.

**Table 2.** Single-year estimated number of PHN cases and associated costs prevented by ZVL in adults aged 50+ years in South Korea

Age, years	No. population^a^	No. individuals with PHN^b^	Incidence rate of PHN, %	Vaccination rate, %	Incidence rate of PHN among the unvaccinated, %	VE of ZVL against PHN^c^, %	Expected number of PHN cases prevented by ZVL	Expected HCS cost savings from PHN cases prevented by ZVL, US dollars	Expected societal cost savings from PHN cases prevented by ZVL, US dollars
	A	B	C = B/A × 100	D	E = C/{(1-F) × D +(1-D)}	F	G = A × D × E × F		
50–54	4525612	17476	0.39	10.00 [70.00]	0.41	59.70	1110 [7767]	1372905 [9610333]	2412102 [16884711]
55–59	4070751	22740	0.56	10.00 [70.00]	0.59	59.70	1444 [10106]	2305632 [16139426]	4302965 [30120757]
60–64	4258205	35060	0.82	10.00 [70.00]	0.88	59.70	2226 [15582]	6740719 [47185030]	11582647 [81078532]
65–69	3274183	40626	1.24	10.00 [70.00]	1.32	59.70	2579 [18056]	4270397 [29892777]	6767458 [47372208]
70–74	2234677	39591	1.77	10.00 [70.00]	1.88	59.70	2514 [17596]	5349610 [37447267]	7516872 [52618102]
75–79	1637098	37124	2.27	10.00 [70.00]	2.41	59.70	2357 [16499]	6679473 [46756308]	9191611 [64341276]
80+	2289858	59202	2.59	10.00 [70.00]	2.75	59.70	3759 [26311]	10579605 [74057235]	17281032 [120967221]
Total	22290384	251819					15988 [111917]	37298339 [261088376]	59054687 [413382806]

a Data source: Korea Statistical Information Service (KOSIS), 2023.

b Data source: Health Insurance Review and Assessment Services (HIRA) Open Statistics in 2023. Diagnosis of PHN was defined according to the International Classification of Diseases Code, 10th version (ICD-10 code) of G53.0.

c Data source: Mbinta JF, et al. (2022) [20].

HCS, healthcare system; PHN, postherpetic neuralgia; VE, vaccine effectiveness; ZVL, live-attenuated vaccine. All cost estimates are reported in US dollars (1 USD = 1300 Korean won). Values in [ ] represent estimates based on the assumed vaccination rate after reaching the target level following the introduction of NIP.

At 10% coverage, RZV would prevent 56681 HZ cases, corresponding to cost savings of USD 18.10 million and USD 30.64 million from the healthcare system and societal perspectives, respectively. When coverage increases to 70% under the NIP, the number of HZ cases prevented by RZV rises to 396764, translating into savings of USD 126.72 million (healthcare system) and USD 214.45 million (societal) (Table 3). Regarding PHN, RZV at 10% coverage would prevent 20712 cases, saving USD 48.32 million (healthcare system) and USD 76.50 million (societal). At 70% coverage, RZV is projected to prevent 144987 PHN cases, with corresponding savings of USD 338.24 million (healthcare system) and USD 535.53 million (societal) (Table 4). RZV generated consistently higher savings across all age groups, with the largest benefits observed in the ≥80 age group.

**Table 3.** Single-year estimated number of HZ cases and associated costs prevented by RZV in adults aged 50+ years in South Korea

Age, years	No. population^a^	No. individuals with HZ^b^	Incidence rate of HZ, %	Vaccination rate^c^, %	Incidence rate of HZ among the unvaccinated, %	VE of RZV (two doses) against HZ^d^, %	Expected number of HZ cases prevented by RZV (two doses)	Expected HCS cost savings from HZ cases prevented by RZV (two doses), US dollars	Expected societal cost savings from HZ cases prevented by RZV (two doses), US dollars
	A	B	C = B/A × 100	D	E = C/{(1-F) × D +(1-D)}	F	G = A × D × E × F		
50–54	4525612	99422	2.20	10.00 [70.00]	2.39	79.20	8552 [59861]	2029305 [14205134]	4323521 [30264644]
55–59	4070751	107994	2.65	10.00 [70.00]	2.88	79.20	9289 [65022]	2432729 [17029100]	4887391 [34221737]
60–64	4258205	130796	3.07	10.00 [70.00]	3.34	79.20	11250 [78750]	3309853 [23168972]	5621836 [39352854]
65–69	3274183	114014	3.48	10.00 [70.00]	3.78	79.20	9807 [68646]	3019551 [21136857]	4833959 [33837715]
70–74	2234677	79558	3.56	10.00 [70.00]	3.87	79.20	6843 [47901]	2300496 [16103474]	3486424 [24404970]
75–79	1637098	57787	3.53	10.00 [70.00]	3.83	79.20	4970 [34793]	1883580 [13185062]	2674602 [18722214]
80+	2289858	69412	3.03	10.00 [70.00]	3.29	79.20	5970 [41792]	3127889 [21895225]	4808084 [33656586]
Total	22290384	658983					56681 [396764]	18103404 [126723825]	30635817 [214450721]

a Data source: Korea Statistical Information Service (KOSIS), 2023.

b Data source: Health Insurance Review and Assessment Services (HIRA) Open Statistics in 2023. Diagnosis of HZ was defined according to the International Classification of Diseases Code, 10th version (ICD-10 code) of B02.

c In the base-case analysis, RZV vaccination coverage refers to completion of the two-dose series (full-series coverage). Completion-rate uncertainty was addressed separately via scenario analysis (Supplementary Appendix Table C5).

d Data source: Mbinta JF, et al. (2022) [20].

HCS, healthcare system; HZ, herpes zoster; VE, vaccine effectiveness; RZV, recombinant zoster vaccine. All cost estimates are reported in US dollars (1 USD = 1300 Korean won). Values in [ ] represent estimates based on the assumed vaccination rate after reaching the target level following the introduction of NIP.

**Table 4.** Single-year estimated number of PHN cases and associated costs prevented by RZV in adults aged 50+ years in South Korea

Age, years	No. population^a^	No. individuals with PHN^b^	Incidence rate of PHN, %	Vaccination rate^c^, %	Incidence rate of PHN among the unvaccinated, %	VE of RZV (two doses) against PHN^d^, %	Expected number of PHN cases prevented by RZV (two doses)	Expected HCS cost savings from PHN cases prevented by RZV (two doses), US dollars	Expected societal cost savings from PHN cases prevented by RZV (two doses), US dollars
	A	B	C = B/A × 100	D	E = C/{(1-F) × D +(1-D)}	F	G = A × D × E × F		
50–54	4525612	17476	0.39	10.00 [70.00]	0.42	76.00	1437 [10062]	1778583 [12450080]	3124851 [21873957]
55–59	4070751	22740	0.56	10.00 [70.00]	0.60	76.00	1870 [13093]	2986921 [20908448]	5574444 [39021109]
60–64	4258205	35060	0.82	10.00 [70.00]	0.89	76.00	2884 [20186]	8732527 [61127687]	15005192 [105036346]
65–69	3274183	40626	1.24	10.00 [70.00]	1.34	76.00	3342 [23391]	5532252 [38725764]	8767168 [61370173]
70–74	2234677	39591	1.77	10.00 [70.00]	1.92	76.00	3256 [22795]	6930360 [48512522]	9738024 [68166171]
75–79	1637098	37124	2.27	10.00 [70.00]	2.45	76.00	3053 [21374]	8653183 [60572282]	11907631 [83353414]
80+	2289858	59202	2.59	10.00 [70.00]	2.80	76.00	4869 [34086]	13705762 [95940333]	22387386 [156711702]
Total	22290384	251819					20712 [144987]	48319588 [338237116]	76504696 [535532873]

a Data source: Korea Statistical Information Service (KOSIS), 2023.

b Data source: Health Insurance Review and Assessment Services (HIRA) Open Statistics in 2023. Diagnosis of PHN was defined according to the International Classification of Diseases Code, 10th version (ICD-10 code) of G53.0.

c In the base-case analysis, RZV vaccination coverage refers to completion of the two-dose series (full-series coverage). Completion-rate uncertainty was addressed separately via scenario analysis (Supplementary Appendix Table C5).

d Data source: Mbinta JF, et al. (2022) [20].

HCS, healthcare system; PHN, postherpetic neuralgia; VE, vaccine effectiveness; RZV, recombinant zoster vaccine. All cost estimates are reported in US dollars (1 USD = 1300 Korean won). Values in [ ] represent estimates based on the assumed vaccination rate after reaching the target level following the introduction of NIP.

### Comparison of preventable HZ and PHN cases and costs by vaccine type (RZV vs. ZVL)

Across all age groups, RZV demonstrated substantially higher preventive effectiveness for both HZ and PHN than ZVL. In the total population aged 50 and older, ZVL was projected to prevent 36269–253880 HZ cases during the one-year study horizon, depending on coverage (Table 1), while RZV could prevent 56681–396764 (Table 3), representing approximately 1.6 times more cases than ZVL. A similar pattern was observed for PHN: ZVL could prevent 15988–111917 cases (Table 2), while RZV could prevent 20712–144987 (Table 4), about 1.3 times more than ZVL.

This superiority of RZV was maintained even among the oldest and highest-burden population. Among adults aged ≥80 years – where ZVL effectiveness is lower – RZV still prevented 1.9 times more HZ cases (5970 vs. 3187) and 1.3 times more PHN cases (4869 vs. 3759) at current coverage levels. With NIP-scale coverage, the absolute difference in prevented cases became more pronounced, with RZV preventing an additional 19482 HZ cases (41792 vs. 22310) and 7775 PHN cases (34086 vs. 26311) compared to ZVL (Tables 1–4).

From the healthcare system perspective, ZVL is projected to save USD 48.60–340.22 million by preventing HZ and PHN (Tables 1 and 2), while RZV is projected to save USD 66.42–464.96 million (Tables 3 and 4), implying a differential of USD 17.82–124.74 million. From the societal perspective, RZV is estimated to save USD 28.67–200.64 million more compared to ZVL, with total cost savings of USD 107.14–749.98 million for RZV (Tables 3 and 4) and USD 78.47–549.34 million for ZVL (Tables 1 and 2).

### Projected cost savings by vaccine market share

**Figure 1.**
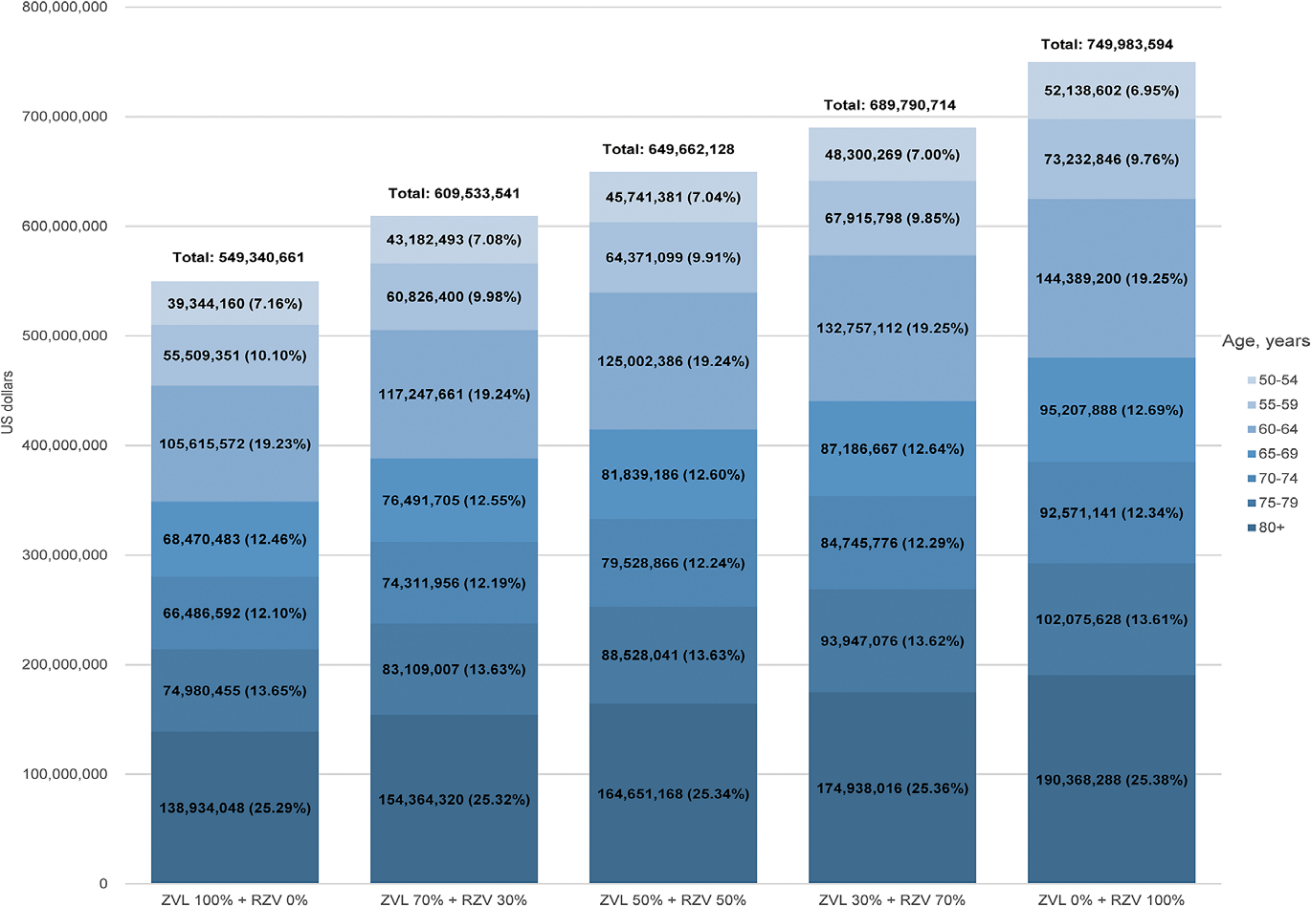
Projected societal cost savings associated with HZ and PHN by varying ZVL and RZV use, assuming 70% uptake under the NIP. This stacked bar chart presents the projected total societal cost savings (in US dollars) associated with HZ and PHN prevention, under five different vaccine market share scenarios: ZVL 100% + RZV 0%, ZVL 70% + RZV 30%, ZVL 50% + RZV 50%, ZVL 30% + RZV 70%, and ZVL 0% + RZV 100%. At 70% vaccine coverage, each bar represents the total societal cost savings among individuals aged ≥50 years with values stratified by seven 5-year age groups: 50–54, 55–59, 60–64, 65–69, 70–74, 75–79, and ≥ 80 years. The absolute cost savings and the percentage contribution of each age group to the total are shown within each bar. Across scenarios, the relative distribution of cost savings by age remained stable (e.g., ≥80 years accounted for 25.29%–25.38% of total savings), while the absolute savings increased as the market share of RZV increased. For example, societal cost savings in the ≥80 age group increased from USD 138.93 million (ZVL 100%) to USD 190.37 million (RZV 100%). The figure included no symbols, annotations, or third-party copyrighted content. It was created by the authors. NIP, National Immunization Program; RZV, recombinant zoster vaccine; ZVL, live-attenuated vaccine. All cost estimates are reported in US dollars (exchange rate: 1 USD = 1300 Korean won).

Assuming a 70% vaccine uptake under the NIP, the projected societal cost savings increased as the market share of RZV grew (Figure 1). Use of ZVL alone (100% market share) was associated with approximately USD 549.34 million in societal cost savings from preventing the incidence of HZ and PHN, whereas RZV alone (100% market share) generated savings of USD 749.98 million. If ZVL and RZV vaccines are used equally with a market share of 50% for each vaccine, cost savings are projected at USD 649.66 million.

Age-stratified analysis indicated that the relative distribution of cost savings across age groups remained stable across market-share scenarios. Across all scenarios, the ≥80 age group consistently accounted for the largest share of total cost savings (25.29% to 25.38%), followed by the 60–64 age group (19.23% to 19.25%). However, while these proportions remained stable, the absolute economic benefits increased as the market share of RZV rose. Specifically, societal savings in the ≥80 age group increased from USD 138.93 million (ZVL 100%) to USD 190.37 million (RZV 100%). Overall, these results suggest that although the age distribution of savings is broadly similar across vaccine-mix scenarios, prioritizing RZV maximizes the absolute financial and public health benefits, particularly in the oldest adults who bear the highest burden of severe disease and costly complications.

### Sensitivity analysis

A sensitivity analysis assessed how varying VE influences economic impact of vaccination. Under the current 10% coverage, ZVL was projected to generate societal cost savings of USD 64.16–90.12 million from prevented HZ and PHN cases. This expanded to USD 449.09–630.81 million at 70% coverage. RZV consistently outperformed ZVL across all scenarios, generating up to USD 117.96 million (current coverage) and USD 825.73 million (70% coverage) in societal cost savings (Table 5). Additionally, in the sensitivity analysis for vaccine coverage, projected cost savings increased linearly as the uptake rate was varied from 50% to 90% (Supplementary Appendix Table C2).

**Table 5.** Sensitivity analysis results of cost savings from a societal perspective by variations in the vaccine effectiveness of ZVL and RZV

Age, years	Applying 95% CI of VE of ZVL^a^		Applying 95% CI of VE of RZV^a^	
	Lower bound	Upper bound	Lower bound	Upper bound
	Cost savings of HZ + PHN cases prevented by ZVL, US dollars	Cost savings of HZ + PHN cases prevented by ZVL, US dollars	Cost savings of HZ + PHN cases prevented by RZV, US dollars	Cost savings of HZ + PHN cases prevented by RZV, US dollars
50–54	4741342 [33189395]	6297014 [44079099]	5861737 [41032120]	8337768 [58364378]
55–59	6619710 [46337968]	8949705 [62647933]	8449068 [59147477]	11636950 [81458653]
60–64	12338471 [86369297]	17342263 [121395840]	17389398 [121725788]	22692941 [158850585]
65–69	8057804 [56404628]	11203195 [78422367]	11261109 [78827762]	15033822 [105236756]
70–74	7728372 [54098603]	10952541 [76667785]	11170189 [78191321]	14541582 [101791074]
75–79	8666638 [60666466]	12381706 [86671944]	12530021 [87710145]	15961458 [111730207]
80+	16003657 [112025596]	22989545 [160926815]	23400916 [163806410]	29756435 [208295046]
Total	64155993 [449091953]	90115969 [630811783]	90062432 [630437023]	117960957 [825726698]

a Data source: Mbinta JF, et al. (2022) [20].

CI, confidence interval; HZ, herpes zoster; PHN, postherpetic neuralgia; RZV, recombinant zoster vaccine; VE, vaccine effectiveness; ZVL, live-attenuated vaccine. All cost estimates are reported in US dollars (1 USD = 1300 Korean won). Values in [ ] represent estimates based on the assumed vaccination rate after reaching the target level following the introduction of NIP.

Sensitivity analyses varying the incidence rates of HZ and PHN by ±20% did not materially change the overall conclusions. Even under the conservative scenario assuming a 20% lower incidence, both ZVL and RZV consistently generated substantial disease-associated cost savings from both the healthcare system and societal perspectives. Detailed results of these scenario analyses are provided in Supplementary Appendix Tables C3 and C4.

Scenario analyses varying the RZV second-dose completion rate showed that projected benefits decreased proportionally as completion declined (Supplementary Appendix Table C5). Under the 70% dose-1 uptake assumption, societal cost savings ranged from USD 374.99 million (50% completion; effective full-series coverage 35%) to USD 749.98 million (100% completion; effective full-series coverage 70%), with corresponding prevented cases of 198382–396764 for HZ and 72493–144987 for PHN.

## Discussion

This study estimated the potential epidemiological and economic impact of introducing ZVL and RZV into the NIP for adults aged ≥50 years in South Korea, assuming expanded vaccination coverage. Our findings indicate that both ZVL and RZV could substantially reduce cases of HZ and PHN, thereby leading to significant cost savings associated with the treatment of these conditions. In particular, RZV consistently demonstrated a greater preventive impact across all analytical scenarios. By estimating preventable cases based on the incidence rate in the unvaccinated population, our approach offers a more precise projection of the NIP’s potential benefits compared to methods using overall population incidence.

The present analysis highlights the preventive potential of both vaccines against HZ and PHN in adults aged ≥50 years. Under current coverage assumptions (10%), ZVL was projected to prevent approximately 36269 HZ cases and 15988 PHN cases, while RZV was estimated to prevent 56681 HZ cases and 20712 PHN cases (Tables 1–4). If coverage increased to 70%, the number of preventable HZ and PHN cases was projected to increase by approximately sevenfold for both vaccines, underscoring the importance of enhancing vaccination coverage to maximize public health benefits. From an economic perspective, reductions in HZ and PHN cases translated into substantial cost savings from both healthcare system and societal perspectives. At 70% coverage, ZVL was estimated to yield cost savings of USD 340.22 million from the healthcare system perspective and USD 549.34 million from the societal perspective, while RZV was projected to generate cost savings of USD 464.96 million and USD 749.98 million, respectively. These differences in averted costs largely reflect the higher real-world effectiveness of RZV compared with ZVL, consistent with observational effectiveness evidence [21, 30] and international cost-effectiveness literature [31, 32]. Based on such comparative evidence and other cost-effectiveness analyses [28, 33], the US Advisory Committee on Immunization Practices (ACIP) currently recommends RZV over ZVL for adults aged ≥50 years [34]. Our findings provide Korea-specific estimates suggesting that greater use of RZV could yield larger health and economic gains than ZVL under the assumptions examined, offering evidence to inform future updates to domestic immunization policies.

Recent Korea-specific studies have evaluated HZ vaccination policies in adults aged ≥50 years, including a predictive public health impact model and a cost-effectiveness analysis [10, 35]. These studies consistently suggest that increasing vaccine uptake could substantially reduce HZ/PHN burden in Korea, and they generally project larger health gains for RZV than for ZVL. However, conclusions regarding economic value differ by analytic framework and assumptions – particularly whether vaccination programme costs are included and what vaccine prices are assumed. For example, the recent Korean cost-effectiveness analysis reported that while RZV yields greater reductions in HZ incidence, ZVL may be more cost-effective in Korea primarily due to its substantially lower vaccine price [10]. Our analysis is complementary to these studies by focusing on a one-year (2025) horizon, updating disease-associated cost inputs to 2025 values, evaluating market-share mix scenarios, and parameterizing vaccine performance using post-licensure real-world effectiveness estimates rather than relying solely on pivotal trial efficacy inputs.

A major strength of our updated analysis is the use of post-licensure real-world effectiveness estimates derived from observational studies, rather than relying solely on efficacy estimates from pivotal randomized trials. Although RCTs provide high internal validity, their controlled settings and strict eligibility criteria may limit generalizability to routine practice (e.g., broader populations, adherence to two-dose series of RZV, and real-world healthcare-seeking behaviour). Observational effectiveness studies may better reflect real-world conditions, although they may be subject to residual confounding; therefore, we assessed uncertainty using confidence-interval-based sensitivity analyses.

Currently, both ZVL and RZV are recommended for adults aged ≥50 years in South Korea [15, 16]. RZV is prioritized due to its greater efficacy and longer duration of protection, while ZVL remains a viable option given its lower cost, favourable safety profile, and contribution to ensuring patient choice [16]. Our scenario analysis examined different market share distributions of ZVL and RZV under the 70% NIP uptake assumption. The results demonstrated that as the share of RZV increased, the total cost savings associated with HZ and PHN prevention also increased compared with a scenario of 100% ZVL use (Figure 1). This suggests that greater use of the vaccine with higher real-world effectiveness could yield larger disease-associated cost savings (i.e., averted medical and societal costs) and greater reductions in HZ/PHN cases, while noting that vaccination programme costs were not included in this analysis.

Despite this favourable outlook and accumulating evidence regarding the safety of both vaccines [19, 21, 36, 37], vaccination coverage in South Korea remains low [10, 17, 18], underscoring the need for effective strategies to enhance uptake. Public education or awareness campaigns that stress the substantial disease burden of HZ and PHN, together with the demonstrated safety, efficacy, durability, and potential economic benefits of both ZVL and RZV, could be instrumental in improving vaccination coverage. Importantly, the high out-of-pocket cost of HZ vaccination has been identified as one of the major barriers to uptake [18]. Reducing financial barriers through measures such as government subsidies or inclusion in the NIP is likely to be critical for improving vaccination coverage.

This study has several limitations related to input parameters and assumptions, which may have led to over- or underestimation. First, the definition of both HZ and PHN cases in this study was broad. Specifically, HZ cases were identified using ICD-10 code B02 from the 2023 HIRA Open Statistics, and PHN cases were defined using ICD-10 code G53.0 [4]. In contrast, previous studies adopted a more conservative approach by utilizing not only these diagnostic codes but also additional criteria such as treatment duration and records of antiviral prescriptions [5, 10, 38]. As a result, our estimates of prevention and associated cost savings may have been overstated. To address the impact of this potential overestimation, we performed additional sensitivity analyses varying the incidence rates by ±20%. Even under the lower-incidence scenario, the introduction of both vaccines continued to produce meaningful disease-associated cost savings, and the comparative conclusion that RZV yields larger health and economic gains than ZVL remained unchanged. These findings suggest that while the absolute magnitude of the estimates is sensitive to case definitions, the main policy-relevant conclusions are stable across plausible incidence ranges. In addition, because our cost inputs were derived from administrative claims analyses reported in prior literature, we could not directly stratify either HZ or PHN costs by patient-level symptom severity (e.g., pain intensity) or clinically observed treatment duration. Instead, differences in disease burden – particularly for PHN – are reflected indirectly through observed healthcare utilization patterns (e.g., more outpatient visits and longer hospitalization days), as summarized in Supplementary Appendix Table C1. Second, projections assumed that healthcare utilization patterns and incidence rates observed in recent years would remain stable through 2025, although these may vary with evolving epidemiology. Furthermore, our model utilized an average annual incidence rate, thereby not capturing the known seasonal variations in HZ occurrence. Several studies, including those in South Korea, have reported that HZ incidence peaks during the summer months [39–41]. This simplification could lead to an underestimation of the disease burden during peak seasons, although the overall impact on the net annual estimates is likely minimal.

Third, the incidence in the unvaccinated population was estimated separately for the ZVL and RZV models, based on the respective VE inputs. In reality, the incidence among unvaccinated individuals should not differ by vaccine type. However, because the VE values for ZVL and RZV differ, the resulting incidence rates also vary between the two models. Fourth, the analysis did not account for prior vaccination history among the target population. Given that HZ vaccines are generally administered as a complete immunization course (typically one or two doses) rather than an annual vaccination, individuals who were vaccinated in earlier years may not be eligible for revaccination within the study period. By assuming that all adults aged ≥50 years had no prior HZ vaccination, our model may have overestimated the number of individuals eligible for vaccination. Moreover, the possibility of HZ recurrence among unvaccinated individuals was not considered. Furthermore, economic evaluations outside of South Korea demonstrated the cost-effectiveness of administering RZV to individuals previously vaccinated with ZVL [42]. Future long-term modelling studies in South Korea should incorporate prior vaccination history and explicitly evaluate transition or booster strategies. Fifth, the model did not incorporate the waning of VE over time, and results were limited to a one-year time horizon. Therefore, the projected number of prevented cases and associated cost savings should be interpreted as short-term annual estimates rather than long-term cumulative effects. Sixth, our base-case RZV analysis assumes completion of the two-dose series because the effectiveness inputs were based on the complete regimen; thus, coverage values for RZV in the base case represent full-series (two-dose) coverage. This assumption is consistent with recent Korea-specific modelling work, which also assumed full compliance with the two-dose RZV schedule in the base case [35]. In practice, second-dose completion may be lower (e.g., 56.2% as noted in U.S. settings) [28, 29], which would reduce population-level benefits. Nevertheless, high completion has been reported in other adult two-dose vaccination programmes in South Korea [43], although this may not directly translate to RZV. We, therefore, evaluated completion-rate scenarios (50%–100% among dose-1 recipients, covering the aforementioned 56.2% estimate [29]) and found that absolute benefits decrease proportionally with lower completion, although substantial reductions in HZ/PHN burden and disease-related cost savings remain even under suboptimal completion (Supplementary Appendix Table C5). Because robust and generalizable one-dose effectiveness estimates were not available for base-case parameterization, we conservatively assumed zero protection for incomplete regimens; future studies should incorporate Korea-specific completion data and one-dose effectiveness evidence when available.

Seventh, our findings on the economic impact of vaccination should be interpreted with caution. The analysis considered only the reduction in disease-associated costs for HZ and PHN, providing a limited estimate, and did not account for net cost savings that would include both medical and vaccination expenses. As previously noted, this exclusion reflects the separate financing structures for disease treatment and the NIP in South Korea, which makes future vaccine procurement prices highly uncertain. Future research should incorporate vaccination expenses and explore plausible procurement price scenarios to estimate the net economic impact.

Finally, VE inputs were derived from our updated evidence synthesis and parameterized using post-licensure real-world effectiveness estimates from Mbinta et al. [20]. To reflect uncertainty, we conducted one-way sensitivity analyses using the lower and upper bounds of the 95% confidence intervals for VE estimates (Table 5). We also examined uncertainty in key programmatic assumptions by varying vaccine coverage from 50% to 90% [24–27] and by conducting additional scenario analyses for incidence definitions and, for RZV, two-dose completion rates (Supplementary Appendix C).

## Conclusion

In conclusion, HZ vaccination in adults aged 50 years and older in South Korea has the potential to prevent a substantial number of HZ and PHN cases while reducing associated healthcare costs. If incorporated into the NIP and coverage increases to 70%, these benefits could expand nearly sevenfold, underscoring the need for interventions to improve vaccine uptake. These findings provide important evidence to inform policy development, programme refinement, and resource allocation decisions regarding HZ vaccination.

## Supporting information

10.1017/S0950268826101502.sm001 Kim et al. supplementary material Kim et al. supplementary material

## Data Availability

All relevant data are presented in the manuscript.

## Supplementary material

The supplementary material for this article can be found at http://doi.org/10.1017/S0950268826101502.
