# Supplementary material for: Epidemiologic and economic impact of introducing a National Immunization Program for herpes zoster vaccination on the prevention of herpes zoster and postherpetic neuralgia in South Korea, an ageing society
Source: Epidemiol Infect. 2026 Apr 30;154:e62. doi: 10.1017/S0950268826101502 (PMC13184655; doi:10.1017/S0950268826101502)
Supplement: Kim et al. supplementary material [file S0950268826101502sup001.docx]

**Supplementary Material**

Appendix A. Calculation steps for HZ and PHN incidence rate among the unvaccinated

Appendix B

Table B1. Search strategies applied in PubMed (Search date: 9 February 2026)

Table B2. Search strategies applied in EMBASE (Search date: 5 February 2026)

Table B3. Search strategies applied in Cochrane Library (Search date: 5 February 2026)

Table B4. Summary of age-specific VE estimates for ZVL and RZV against HZ and PHN from systematic review studies

Table B5. Methodological quality assessment of included systematic reviews using AMSTAR-2

Figure B1. Flowchart for identifying relevant studies

Appendix C

Table C1. Model inputs for the base-case, sensitivity analysis

Table C2. Sensitivity analysis results for variations in the vaccination rate of ZVL and RZV

Table C3. Sensitivity analysis results for variations in the Incidence rate of HZ

Table C4. Sensitivity analysis results for variations in the Incidence rate of PHN

Table C5. Scenario analysis of RZV two-dose completion (assuming 70% dose-1 uptake) and resulting effective full-series coverage.

**Appendix** A. Calculation steps for HZ and PHN incidence rate among the unvaccinated

$$Incidence rate in the vaccinated$$

$$= Incidence rate in the unvaccinated * (1-VE against HZ (or \mathrm{PHN}))$$

*Overall* incidence rate

= Incidence rate in the vaccinated * % vaccinated

+ Incidence rate in the unvaccinated * (1 - % vaccinated)

= Incidence rate in the unvaccinated * (1 - VE against HZ (or PHN)) * % vaccinated

+ Incidence rate in the unvaccinated * (1 - % vaccinated)

Incidence rate *in the unvaccinated*

= Overall incidence rate / {(1 - VE against HZ (or PHN)) * % vaccinated + (1 - % vaccinated)}

*HZ, herpes zoster; PHN, postherpetic neuralgia; VE, vaccine effectiveness

**Appendix B**

**Table B1.** Search strategies applied in PubMed (Search date: 9 February 2026)

| Search No | Query | Hit |
| --- | --- | --- |
| #1 | "Herpes Zoster"[Mesh] OR "Herpes Zoster"[tiab] OR shingles[tiab] OR "Herpesvirus 3, Human"[Mesh] OR "Varicella Zoster Virus"[tiab] | 27,108 |
| #2 | "Herpes Zoster Vaccine"[Mesh] OR "zoster vaccine"[tiab] OR Shingrix[tiab] OR Zostavax[tiab] OR "recombinant zoster vaccine"[tiab] OR "live attenuated zoster vaccine"[tiab] OR RZV[tiab] OR ZVL[tiab] OR "HZ/su"[tiab] | 2,004 |
| #3 | "Vaccine Efficacy"[Mesh] OR "vaccine effectiveness"[tiab] OR efficacy[tiab] OR effectiveness[tiab] OR immunogenicity[tiab] | 2,043,948 |
| #4 | "Systematic Review"[Publication Type] OR "Meta-Analysis"[Publication Type] OR "systematic review"[tiab] OR "meta-analysis"[tiab] OR "network meta-analysis"[tiab] | 573,906 |
| #5 | ("2020/01/01"[Date - Publication] : "3000/12/31"[Date - Publication]) | 9,635,754 |
| #6 | #1 AND #2 AND #3 AND #4 AND #5 | 27 |

**Table B2.** Search strategies applied in EMBASE (Search date: 5 February 2026)

| Search No | Query | Hit |
| --- | --- | --- |
| #1 | herpes zoster'/exp OR 'herpes zoster':ti,ab,kw OR 'shingles':ti,ab,kw OR 'varicella zoster virus'/exp | 54,344 |
| #2 | herpes zoster vaccine'/exp OR 'zoster vaccine live'/exp OR 'recombinant zoster vaccine'/exp OR 'zostavax':ti,ab,kw OR 'shingrix':ti,ab,kw OR 'live attenuated zoster vaccine':ti,ab,kw OR 'recombinant subunit vaccine':ti,ab,kw OR 'RZV':ti,ab,kw OR 'ZVL':ti,ab,kw OR 'HZ/su':ti,ab,kw | 6,097 |
| #3 | vaccine efficacy'/exp OR 'vaccine effectiveness'/exp OR 'efficacy':ti,ab,kw OR 'effectiveness':ti,ab,kw OR 'immunogenicity':ti,ab,kw | 3,473,777 |
| #4 | systematic review'/exp OR 'meta analysis'/exp OR 'systematic review':ti,ab,kw OR 'meta analysis':ti,ab,kw OR 'medline':ti,ab,kw OR 'pubmed':ti,ab,kw OR 'embase':ti,ab,kw | 1,013,023 |
| #5 | #1 AND #2 AND #3 AND #4 | 263 |
| #6 | [2020-2026]/py | 11,831,944 |
| #7 | #5 AND #6 | 144 |

**Table B3.** Search strategies applied in Cochrane Library (Search date: 5 February 2026)

| Search No | Query | Hit |
| --- | --- | --- |
| #1 | MeSH descriptor: [Herpes Zoster] explode all trees | 821 |
| #2 | (herpes zoster):ti,ab,kw OR (shingles):ti,ab,kw OR (varicella zoster virus):ti,ab,kw | 3,131 |
| #3 | #1 OR #2 | 3,131 |
| #4 | MeSH descriptor: [Herpes Zoster Vaccine] explode all trees | 139 |
| #5 | ((zoster NEXT vaccine*) OR shingrix OR zostavax OR "recombinant zoster vaccine" OR "live attenuated zoster vaccine" OR RZV OR ZVL OR "HZ/su"):ti,ab,kw | 406 |
| #6 | #4 OR #5 | 406 |
| #7 | (efficacy):ti,ab,kw OR (effectiveness):ti,ab,kw OR (immunogenicity):ti,ab,kw | 676,931 |
| #8 | (systematic review):ti,ab,kw OR (meta-analysis):ti,ab,kw OR (network meta-analysis):ti,ab,kw | 30,782 |
| #9 | #3 AND #6 AND #7 AND #8 with Cochrane Library publication date Between Jan 2020 and Dec 2026 | 5 |

**Table B4.** Summary of age-specific VE estimates for ZVL and RZV against HZ and PHN from systematic review studies

| First Author, Year | Mbinta et al. (2022) [1] | Xia et al. (2022) [2] | Zeevaert et al. (2023) [3] |
| --- | --- | --- | --- |
| Study design | Systematic review and meta-analysis | Systematic review and meta-analysis, network meta-analyses | Systematic review and meta-analysis |
| Search period | 25 May 2006–31 December 2020 (Updated 31 January 2021) | Up to January 2022 | from inception data until 7 February 2022. |
| Databases | MEDLINE, Embase, Cochrane, CINAHL, ProQuest, Dimensions, Grey literature | Web of Science, PubMed, Embase, Cochrane Library | OVID MEDLINE, Embase and Cochrane CENTRAL |
| Included studies (RCTs/Obs) | 22 Observational Studies (21 Cohort, 1 Case-control) | 36 Studies (17 RCTs, 19 Cohort) | 14 Studies (23 Publications) (6 RCTs, 8 Observational) |
| Target population^a^ | Adults ≥50 years (Immunocompetent & Immunocompromised) | Adults (Immunocompetent ≥50 years & Immunocompromised) | Immunocompetent ≥50 years & Immunocompromised ≥18 years |
| Sample size | > 25 million (Cumulative) (9.5M for ZVL + 15.5M for RZV) | ~ 84,264 (80,980 Immunocompetent + 3,284 Immunocompromised) Observational: Large cohorts (millions of person-years) | RCTs: ~32,200^b^ Observational: > 20 million |
| Primary outcomes | VE against HZ, PHN, HZO | Efficacy/Effectiveness against HZ, PHN, HZO | QoL, Reactogenicity, Safety, Efficacy/Effectiveness against HZ, PHN, HZO |
| VE estimates for ZVL: HZ (95% CI)  (Immunocompetent) | Effectiveness (RWD): 50–59y: 60.0% (53.0–65.0) 60–69y: 50.9% (45.0–56.1) 70–79y: 46.6% (40.7–51.9) ≥80y: 43.9% (37.7–49.5) Overall: 44.7% (38.4–50.3) | Effectiveness (RWD): Overall: 42% (30–52)  Efficacy (RCT): Overall: 62% (23–82) | N/A |
| VE estimates for ZVL: PHN (95% CI)  (Immunocompetent) | Effectiveness (RWD): Overall: 59.7% (48.3–68.7) | Effectiveness (RWD): Overall: 63% (36–79)  Efficacy (RCT): Overall: 66.5% (47.5–79.2) | N/A |
| VE estimates for RZV: HZ (95% CI)  (Immunocompetent) | Effectiveness (RWD): Overall: 79.2% (57.6–89.7) (post-hoc analysis, 2 studies) [two doses] | Effectiveness (RWD): Overall: 70% (56–80) [two doses]  Efficacy (RCT): Overall: 94% (87–97) [two doses] | Efficacy (RCT): ≥50y: 94% (79–98.0) ≥70y: 91.3% (86.8–94.5) [two doses]  Effectiveness (RWD): Overall: 70.5 – 85.0% [two doses] |
| VE estimates for RZV: PHN (95% CI)  (Immunocompetent) | Effectiveness (RWD): Overall: 76.0% (68.4–81.8) [two doses] | Effectiveness (RWD): Overall: 77% (34–92%) [unclear]^c^  Efficacy (RCT): ≥50y: 91.2% (75.9–97.7) ≥70y: 88.8% (68.7–97.1) [two doses] | Effectiveness (RWD):  ≥65 years: 76% (68–82) (1 study) [two doses]  Efficacy (RCT): ≥50y: 91.2% (75.9–97.7) ≥70y: 88.8% (68.7–97.1) [two doses] |
| Quality assessment tool | JBI Critical Appraisal Checklist | Cochrane Risk of Bias Tool | Cochrane Risk of Bias Tool 2, ROBINS-I, GRADE |
| Use in current model^d^ | base-case analysis | Referenced only | Referenced only |
| CI, confidence interval; GRADE, Grading of Recommendations Assessment, Development and Evaluation; HZ, herpes zoster; HZO, herpes zoster ophthalmicus; JBI, Joanna Briggs Institute; Obs, observational studies; PHN, postherpetic neuralgia; QoL, quality of life; RCT, randomized controlled trial; ROBINS-I, Risk Of Bias In Non-randomized Studies - of Interventions; RWD, real-world data; RZV, recombinant zoster vaccine; VE, vaccine effectiveness; ZVL, live-attenuated vaccine. ^a^Although the included reviews covered both immunocompetent and immunocompromised populations, the VE estimates presented in this table and extracted for the model specifically refer to the immunocompetent population. ^b^Zeevaert et al. does not report a single aggregated RCT sample size as “32,200.” The value shown here is an approximate figure derived by summing the RCT populations reported in the review (e.g., ZOE-50, ZOE-70, and additional RCTs) and rounding for presentation. ^c^Xia et al. reported “R-1ZV” with 77% effectiveness (95% CI: 34%–92%) for PHN prevention in the effectiveness network meta-analysis; however, “R-1ZV” was not explicitly defined as a standard vaccine/dosing term in the manuscript, and the regimen corresponding to this estimate is therefore unclear. ^d^Mbinta et al. was selected as the base-case data source because it provided age stratification (50–59, 60–69, 70–79, ≥80 years) necessary for age-cohort Markov modeling of herpes zoster incidence and vaccine effectiveness. Xia et al. and Zeevaert et al. were used for contextual comparison of efficacy/effectiveness evidence and were not parameterized in the model.  [1] **Mbinta JF, *et al.*** (2022) Post-licensure zoster vaccine effectiveness against herpes zoster and postherpetic neuralgia in older adults: a systematic review and meta-analysis. *The Lancet Healthy Longevity*; **3**: e263–e275. doi:10.1016/S2666-7568(22)00039-3. [2] **Xia Y, *et al.*** (2022) Efficacy, effectiveness, and safety of herpes zoster vaccine in the immunocompetent and immunocompromised subjects: A systematic review and network meta-analysis. *Frontiers in Immunology*; **13**: 978203. doi:10.3389/fimmu.2022.978203. [3] **Zeevaert R, *et al.*** (2023) Efficacy and safety of the recombinant zoster vaccine: A systematic review and meta-analysis. *Vaccine X*; **15**: 100397. doi:10.1016/j.jvacx.2023.100397. | | | |

**Table B5.** Methodological quality assessment of included systematic reviews using AMSTAR-2

| Item (AMSTAR-2 Critical Domains) | Mbinta et al. (2022) [1] | Xia et al. (2022) [2] | Zeevaert et al. (2023) [3] |
| --- | --- | --- | --- |
| 2. Protocol registered before review? | Yes (PROSPERO, CRD42021232383) | Yes (PROSPERO, CRD42022310495) | Yes (PROSPERO, CRD42022311749) |
| 4. Comprehensive literature search? | Yes (6 databases + grey lit) | Yes (4 databases + grey lit) | Yes (3 databases + grey lit) |
| 7. List of excluded studies provided? | Yes (supplementary p.30-32**)** | Yes (Appendix S2) | Yes (supplementary material Table S1) |
| 9. Risk of bias assessed? | Yes (JBI Critical Appraisal Checklist) | Yes (Cochrane Risk of Bias Tool) | Yes (Cochrane Risk of Bias Tool 2, ROBINS-I, GRADE) |
| 11. Appropriate meta-analysis methods? | Yes (Random effects) | Yes (Random/fixed, network meta-analyses) | Yes (Random effects) |
| 13. Risk of bias accounted for in interpretation? | Yes | Partial Yes^a^ | Yes |
| 15. Publication bias assessed? | Partial Yes^b^ | Yes | No^c^ |
| **Overall Confidence Rating** | **High** | **High** | **Moderate** |
| AMSTAR-2, Assessing the Methodological Quality of Systematic Reviews 2; GRADE, Grading of Recommendations Assessment; JBI, Joanna Briggs Institute; ROBINS-I, Risk Of Bias In Non-randomized Studies - of Interventions; ^a^Risk of bias was assessed and reported in Appendix S4-6. However, discussion of its impact on pooled results was limited. ^b^Publication bias was considered but could not be formally assessed. supplementary Table S7 footnote states: “A funnel plot could not be used to assess publication bias since less than 10 studies were included in the meta-analysis.” ^c^Publication bias assessment (funnel plots or statistical tests) was not reported in main text or supplementary materials.  [1] **Mbinta JF, *et al.*** (2022) Post-licensure zoster vaccine effectiveness against herpes zoster and postherpetic neuralgia in older adults: a systematic review and meta-analysis. *The Lancet Healthy Longevity*; **3**: e263–e275. doi:10.1016/S2666-7568(22)00039-3. [2] **Xia Y, *et al.*** (2022) Efficacy, effectiveness, and safety of herpes zoster vaccine in the immunocompetent and immunocompromised subjects: A systematic review and network meta-analysis. *Frontiers in Immunology*; **13**: 978203. doi:10.3389/fimmu.2022.978203. [3] **Zeevaert R, *et al.*** (2023) Efficacy and safety of the recombinant zoster vaccine: A systematic review and meta-analysis. *Vaccine X*; **15**: 100397. doi:10.1016/j.jvacx.2023.100397. | | | |

**Identification of studies through databases and registers**

Records identified from **PubMed**: 27

Records identified from **Embase**: 144

Records identified from **Cochrane Library**: 5

**Identification**

26 Records removed duplicates

150 Records excluded with reasons:

24 Not a systematic review or meta-analysis

9 Not related to ZVL or RZV

38 Study population was not immunocompetent adults aged ≥50 years

69 Did not report VE

**1^st^ exclusion**

10 Pooled Records after 1^st^ exclusion

7 Records excluded with reasons:

0 Not a systematic review or meta-analysis

2 Did not report quantitative VE or efficacy estimates in percentages (%) suitable for model parameterization

3 VE values not reported specifically for adults aged ≥50 years

2 Full text was not available

**2^nd^ exclusion**

**Included**

3 Studies included in reviews

**Figure B1.** Flowchart for identifying relevant studies

RZV, recombinant zoster vaccine; VE, vaccine effectiveness; ZVL, live-attenuated vaccine.

**Appendix C**

**Table C1.** Model inputs for the base-case and sensitivity analysis

|  | Age, years | Base value | Ranges for SA | | | Data source |
| --- | --- | --- | --- | --- | --- | --- |
|  |  |  | Lower bound | | Upper bound |  |
| **Demographics** | | | | | | |
| Population size | 50–54 | 4,525,612 | N/A | | | [1] |
|  | 55–59 | 4,070,751 |  |  |  |  |
|  | 60–64 | 4,258,205 |  |  |  |  |
|  | 65–69 | 3,274,183 |  |  |  |  |
|  | 70–74 | 2,234,677 |  |  |  |  |
|  | 75–79 | 1,637,098 |  |  |  |  |
|  | ≥80 | 2,289,858 |  |  |  |  |
| **Epidemiology** | | | | | | |
| Incidence rate, % | | | | | | |
| HZ | 50–54 | 2.20 | 1.76 | | 2.64 | [2]^a^ |
|  | 55–59 | 2.65 | 2.12 | | 3.18 |  |
|  | 60–64 | 3.07 | 2.46 | | 3.69 |  |
|  | 65–69 | 3.48 | 2.79 | | 4.18 |  |
|  | 70–74 | 3.56 | 2.85 | | 4.27 |  |
|  | 75–79 | 3.53 | 2.82 | | 4.24 |  |
|  | ≥80 | 3.03 | 2.43 | | 3.64 |  |
| PHN | 50–54 | 0.39 | 0.31 | | 0.46 | [2]^b^ |
|  | 55–59 | 0.56 | 0.45 | | 0.67 |  |
|  | 60–64 | 0.82 | 0.66 | | 0.99 |  |
|  | 65–69 | 1.24 | 0.99 | | 1.49 |  |
|  | 70–74 | 1.77 | 1.42 | | 2.13 |  |
|  | 75–79 | 2.27 | 1.81 | | 2.72 |  |
|  | ≥80 | 2.59 | 2.07 | | 3.10 |  |
| **Vaccination parameters** | | | | | | |
| VE of ZVL, % | | | | | | |
| HZ | 50–54 | 60.00 | 53.00 | | 65.00 | [3] |
|  | 55–59 | 60.00 | 53.00 | | 65.00 |  |
|  | 60–64 | 50.90 | 45.00 | | 56.10 |  |
|  | 65–69 | 50.90 | 45.00 | | 56.10 |  |
|  | 70–74 | 46.60 | 40.70 | | 51.90 |  |
|  | 75–79 | 46.60 | 40.70 | | 51.90 |  |
|  | ≥80 | 43.90 | 37.70 | | 49.50 |  |
| PHN | All ages | 59.70 | 48.30 | | 68.70 |  |
| VE of RZV (two doses), % | | | | | | |
| HZ | All ages | 79.20 | 57.60 | | 89.70 | [3] |
| PHN | All ages | 76.00 | 68.40 | | 81.80 |  |
| Vaccination rate, % | | | | | | |
| Current vaccination rate | All ages | 10.00 | N/A | | | [4–6] |
| Expected uptake under NIP | All ages | 70.00 | 50.00 | | 90.00 | [4, 6] |
| RZV two-dose series completion rate, % | All ages | 100.00 | 50.00 | | 100.00 | [7] |
| **Cost and resource use** | | | |  | | |
| Direct medical costs^c^ (Healthcare system perspective), KRW | | | | | | |
| HZ | 50–54 | 308,495 | N/A | | | [6] |
|  | 55–59 | 340,469 |  |  |  |  |
|  | 60–64 | 382,470 |  |  |  |  |
|  | 65–69 | 400,283 |  |  |  |  |
|  | 70–74 | 437,040 |  |  |  |  |
|  | 75–79 | 492,649 |  |  |  |  |
|  | ≥80 | 681,083 |  |  |  |  |
| PHN | 50–54 | 1,608,547 |  |  |  |  |
|  | 55–59 | 2,076,037 |  |  |  |  |
|  | 60–64 | 3,936,677 |  |  |  |  |
|  | 65–69 | 2,152,284 |  |  |  |  |
|  | 70–74 | 2,766,694 |  |  |  |  |
|  | 75–79 | 3,684,027 |  |  |  |  |
|  | ≥80 | 3,659,052 |  |  |  |  |
| Healthcare resource utilization | | | | | | |
| HZ | | | | | | |
| Hospitalization rate, % | 50–54 | 3.31 | N/A | | | [6] |
|  | 55–59 | 4.05 |  |  |  |  |
|  | 60–64 | 4.48 |  |  |  |  |
|  | 65–69 | 4.53 |  |  |  |  |
|  | 70–74 | 4.90 |  |  |  |  |
|  | 75–79 | 5.80 |  |  |  |  |
|  | ≥80 | 9.84 |  |  |  |  |
| Mean No. outpatient visits per case | 50–54 | 2.54 |  |  |  |  |
|  | 55–59 | 2.64 |  |  |  |  |
|  | 60–64 | 2.79 |  |  |  |  |
|  | 65–69 | 3.00 |  |  |  |  |
|  | 70–74 | 3.21 |  |  |  |  |
|  | 75–79 | 3.31 |  |  |  |  |
|  | ≥80 | 3.25 |  |  |  |  |
| Mean hospitalization days per case | 50–54 | 0.32 |  |  |  |  |
|  | 55–59 | 0.40 |  |  |  |  |
|  | 60–64 | 0.49 |  |  |  |  |
|  | 65–69 | 0.52 |  |  |  |  |
|  | 70–74 | 0.59 |  |  |  |  |
|  | 75–79 | 0.77 |  |  |  |  |
|  | ≥80 | 1.73 |  |  |  |  |
| PHN | | | | | | |
| Hospitalization rate, % | 50–54 | 5.70 | N/A | | | [6] |
|  | 55–59 | 7.00 |  |  |  |  |
|  | 60–64 | 8.68 |  |  |  |  |
|  | 65–69 | 9.23 |  |  |  |  |
|  | 70–74 | 9.45 |  |  |  |  |
|  | 75–79 | 10.99 |  |  |  |  |
|  | ≥80 | 16.24 |  |  |  |  |
| Mean No. outpatient visits per case | 50–54 | 9.17 |  |  |  |  |
|  | 55–59 | 11.92 |  |  |  |  |
|  | 60–64 | 32.26 |  |  |  |  |
|  | 65–69 | 15.96 |  |  |  |  |
|  | 70–74 | 17.06 |  |  |  |  |
|  | 75–79 | 27.69 |  |  |  |  |
|  | ≥80 | 28.61 |  |  |  |  |
| Mean hospitalization days per case | 50–54 | 1.03 |  |  |  |  |
|  | 55–59 | 2.64 |  |  |  |  |
|  | 60–64 | 4.58 |  |  |  |  |
|  | 65–69 | 2.66 |  |  |  |  |
|  | 70–74 | 2.73 |  |  |  |  |
|  | 75–79 | 4.38 |  |  |  |  |
|  | ≥80 | 9.85 |  |  |  |  |
| Direct non-medical costs (Societal perspective), KRW | | | | | | |
| Round-trip transportation cost | 50–54 | 5,900 | N/A | | | [8] |
|  | 55–59 | 5,900 |  |  |  |  |
|  | 60–64 | 5,900 |  |  |  |  |
|  | 65–69 | 3,000 |  |  |  |  |
|  | 70–74 | 3,000 |  |  |  |  |
|  | 75–79 | 3,000 |  |  |  |  |
|  | ≥80 | 3,000 |  |  |  |  |
| Caregiving cost | All ages | 120,000 |  |  |  | [9] |
| Indirect cost parameter (Societal perspective) | | | | | | |
| Employment rate, % | 50–54 | 78.80 | N/A | | | [10] |
|  | 55–59 | 75.80 |  |  |  |  |
|  | 60–64 | 65.20 |  |  |  |  |
|  | 65–69 | 54.00 |  |  |  |  |
|  | 70–74 | 42.50 |  |  |  |  |
|  | 75–79 | 27.50 |  |  |  |  |
|  | ≥80 | 27.50 |  |  |  |  |
| Daily wage, KRW | 50–54 | 258,240 |  |  |  | [11] |
|  | 55–59 | 234,275 |  |  |  |  |
|  | 60–64 | 169,042 |  |  |  |  |
|  | 65–69 | 169,042 |  |  |  |  |
|  | 70–74 | 169,042 |  |  |  |  |
|  | 75–79 | 169,042 |  |  |  |  |
|  | ≥80 | 169,042 |  |  |  |  |
| Working days per month | 50–54 | 20.26 |  |  |  | [11] |
|  | 55–59 | 20.36 |  |  |  |  |
|  | 60–64 | 20.21 |  |  |  |  |
|  | 65–69 | 20.21 |  |  |  |  |
|  | 70–74 | 20.21 |  |  |  |  |
|  | 75–79 | 20.21 |  |  |  |  |
|  | ≥80 | 20.21 |  |  |  |  |
| HZ, herpes zoster; KRW, Korean won; N/A, not applicable; NIP, National Immunization Program; PHN, postherpetic neuralgia; RZV, recombinant zoster vaccine; SA, sensitivity analysis; VE, vaccine effectiveness; ZVL, live-attenuated vaccine. ^a^Diagnosis of HZ was defined according to International Classification of Disease Code, 10th version (ICD-10 code) of B02. ^b^Diagnosis of PHN was defined according to International Classification of Disease Code, 10th version (ICD-10 code) of G53.0. ^c^Direct medical costs are presented in 2025 KRW, adjusted using the National Health Insurance Service Medical Cost Conversion Index.  [1] **Korea Statistical Information Service (KOSIS)** (<https://kosis.kr/search/search.do>). Accessed 22 July 2025.  [2] **Health Insurance Review and Assessment Service (HIRA) Open Statistics.** (<https://opendata.hira.or.kr/op/opc/olap3thDsInfoTab1.do>). Accessed 22 July 2025.  [3] **Mbinta JF, *et al.*** (2022) Post-licensure zoster vaccine effectiveness against herpes zoster and postherpetic neuralgia in older adults: a systematic review and meta-analysis. *The Lancet Healthy Longevity*; **3**: e263–e275. doi:10.1016/S2666-7568(22)00039-3.  [4] **Choi WS, *et al.*** (2019) Cost-effectiveness analysis of introducing the zoster vaccine into national immunization program in the Republic of Korea. KDCA Policy Research Report. Korea Disease Control and Prevention Agency.  [5] **Roh NK, *et al.*** (2015) awareness, knowledge, and vaccine acceptability of herpes zoster in Korea: a multicenter survey of 607 patients. *Annals of Dermatology*; **27**: 531–538. doi:10.5021/ad.2015.27.5.531.  [6] **Cheong C, *et al.*** (2025) Cost-effectiveness of including herpes zoster vaccines into the national immunization program in the Republic of Korea. *Vaccine;* **63**: 127648. doi:10.1016/j.vaccine.2025.127648  [7] **Assumption.** The base-case analysis assumes 100% adherence to the two doses RZV schedule. The lower bound (50%) for sensitivity analysis was informed by: **Le P, Rothberg MB.** (2018) Cost-effectiveness of the adjuvanted herpes zoster subunit vaccine in older adults. *JAMA Internal Medicine* **178:** 248–258.  [8] **Seoul Metropolitan Government** (<https://news.seoul.go.kr/traffic>). Accessed 22 July 2025.  [9] **Private Caregiving Services Market Price (Hncare)** ([http://www.hncare.co.kr/Info/Price](http://www.hncare.co.kr/Info/Price%20)). Accessed 22 July 2025.  [10] **Korea Statistical Information Service (KOSIS)** (<https://kosis.kr/statHtml/statHtml.do?sso=ok&returnurl=https%3A%2F%2Fkosis.kr%3A443%2FstatHtml%2FstatHtml.do%3FtblId%3DDT_1DA7002S%26orgId%3D101%26>). Accessed 22 July 2025.  [11] **Korea Statistical Information Service (KOSIS)** (<https://kosis.kr/statHtml/statHtml.do?sso=ok&returnurl=https%3A%2F%2Fkosis.kr%3A443%2FstatHtml%2FstatHtml.do%3FtblId%3DDT_1DA7002S%26orgId%3D101%27>). Accessed 22 July 2025. | | | | | | |

**Table C2.** Sensitivity analysis results for variations in the vaccination rate of ZVL and RZV

| Vaccination rate, % | Age, years | ZVL | | | | RZV | | | |
| --- | --- | --- | --- | --- | --- | --- | --- | --- | --- |
|  |  | Expected number of HZ cases prevented by ZVL | Expected number of PHN cases prevented by ZVL | Expected HCS cost savings from HZ+PHN cases prevented by ZVL, US dollars | Expected societal cost savings from HZ+PHN cases prevented by ZVL, US dollars | Expected number of HZ cases prevented by RZV (two doses) | Expected number of PHN cases prevented by RZV (two doses) | Expected HCS cost savings from HZ+PHN cases prevented by RZV (two doses), US dollars | Expected societal cost savings from HZ+PHN cases prevented by RZV (two doses), US dollars |
| 50.00% | 50–54 | 31,730 | 5,548 | 14,394,278 | 28,102,972 | 42,758 | 7,187 | 19,039,439 | 37,241,858 |
|  | 55–59 | 34,466 | 7,219 | 20,554,823 | 39,649,536 | 46,444 | 9,352 | 27,098,249 | 52,309,176 |
|  | 60–64 | 35,073 | 11,130 | 44,022,285 | 75,439,695 | 56,250 | 14,419 | 60,211,899 | 103,135,143 |
|  | 65–69 | 30,573 | 12,897 | 30,765,640 | 48,907,488 | 49,033 | 16,708 | 42,759,015 | 68,005,634 |
|  | 70–74 | 19,443 | 12,568 | 33,284,505 | 47,490,423 | 34,215 | 16,282 | 46,154,283 | 66,122,244 |
|  | 75–79 | 14,122 | 11,785 | 38,749,226 | 53,557,468 | 24,852 | 15,267 | 52,683,817 | 72,911,163 |
|  | 80+ | 15,936 | 18,794 | 61,246,799 | 99,238,606 | 29,851 | 24,347 | 84,168,256 | 135,977,349 |
|  | Total | 181,343 | 79,940 | 243,017,558 | 392,386,186 | 283,403 | 103,562 | 332,114,958 | 535,702,567 |
| 60.00% | 50–54 | 38,077 | 6,657 | 17,273,134 | 33,723,566 | 51,309 | 8,625 | 22,847,326 | 44,690,230 |
|  | 55–59 | 41,359 | 8,663 | 24,665,788 | 47,579,444 | 55,733 | 11,222 | 32,517,898 | 62,771,011 |
|  | 60–64 | 42,087 | 13,356 | 52,826,743 | 90,527,634 | 67,500 | 17,302 | 72,254,279 | 123,762,171 |
|  | 65–69 | 36,687 | 15,476 | 36,918,768 | 58,688,986 | 58,840 | 20,049 | 51,310,818 | 81,606,761 |
|  | 70–74 | 23,332 | 15,082 | 39,941,407 | 56,988,507 | 41,058 | 19,538 | 55,385,140 | 79,346,693 |
|  | 75–79 | 16,947 | 14,142 | 46,499,072 | 64,268,961 | 29,822 | 18,321 | 63,220,581 | 87,493,396 |
|  | 80+ | 19,123 | 22,553 | 73,496,159 | 119,086,327 | 35,822 | 29,217 | 101,001,907 | 163,172,819 |
|  | Total | 217,612 | 95,928 | 291,621,069 | 470,863,424 | 340,083 | 124,274 | 398,537,950 | 642,843,081 |
| 80.00% | 50–54 | 50,769 | 8,876 | 23,030,845 | 44,964,755 | 68,412 | 11,499 | 30,463,102 | 59,586,973 |
|  | 55–59 | 55,146 | 11,550 | 32,887,717 | 63,439,258 | 74,310 | 14,963 | 43,357,198 | 83,694,681 |
|  | 60–64 | 56,116 | 17,808 | 70,435,657 | 120,703,511 | 90,000 | 23,070 | 96,339,039 | 165,016,228 |
|  | 65–69 | 48,916 | 20,635 | 49,225,023 | 78,251,981 | 78,453 | 26,732 | 68,414,424 | 108,809,015 |
|  | 70–74 | 31,109 | 20,109 | 53,255,209 | 75,984,676 | 54,744 | 26,051 | 73,846,853 | 105,795,590 |
|  | 75–79 | 22,596 | 18,856 | 61,998,762 | 85,691,948 | 39,763 | 24,428 | 84,294,107 | 116,657,861 |
|  | 80+ | 25,497 | 30,070 | 97,994,879 | 158,781,769 | 47,762 | 38,955 | 134,669,209 | 217,563,758 |
|  | Total | 290,149 | 127,905 | 388,828,092 | 627,817,898 | 453,444 | 165,699 | 531,383,933 | 857,124,107 |
| 90.00% | 50–54 | 57,115 | 9,986 | 25,909,700 | 50,585,349 | 76,964 | 12,937 | 34,270,990 | 67,035,345 |
|  | 55–59 | 62,039 | 12,994 | 36,998,682 | 71,369,166 | 83,599 | 16,834 | 48,776,848 | 94,156,516 |
|  | 60–64 | 63,131 | 20,034 | 79,240,114 | 135,791,450 | 101,250 | 25,954 | 108,381,419 | 185,643,257 |
|  | 65–69 | 55,031 | 23,214 | 55,378,151 | 88,033,479 | 88,259 | 30,074 | 76,966,227 | 122,410,142 |
|  | 70–74 | 34,998 | 22,623 | 59,912,110 | 85,482,761 | 61,587 | 29,308 | 83,077,709 | 119,020,039 |
|  | 75–79 | 25,420 | 21,213 | 69,748,607 | 96,403,442 | 44,733 | 27,481 | 94,830,871 | 131,240,094 |
|  | 80+ | 28,684 | 33,829 | 110,244,239 | 178,629,490 | 53,732 | 43,825 | 151,502,861 | 244,759,228 |
|  | Total | 326,418 | 143,893 | 437,431,604 | 706,295,136 | 510,125 | 186,411 | 597,806,924 | 964,264,621 |
| HCS, healthcare system; HZ, herpes zoster; PHN, postherpetic neuralgia; RZV, recombinant zoster vaccine; ZVL, live-attenuated vaccine. All cost estimates are reported in US dollars (1 USD = 1,300 Korean won). | | | | | | | | | |

**Table C3.** Sensitivity analysis results for variations in the incidence rate of HZ

| Vaccine | Age, years | HZ incidence scenario | Expected number of HZ cases | Expected HCS cost savings from HZ cases, US dollars | Expected societal cost savings from HZ cases, US dollars |
| --- | --- | --- | --- | --- | --- |
| ZVL | 50–54 | Low (−20%) | 5,077 [35,538] | 1,204,761 [8,443,325] | 2,566,794 [17,967,559] |
|  |  | Base | 6,346 [44,423] | 1,505,951 [10,541,657] | 3,208,493 [22,459,449] |
|  |  | High (+20%) | 7,615 [53,307] | 1,807,141 [12,649,988] | 3,850,191 [26,951,339] |
|  | 55–59 | Low (−20%) | 5,515 [38,602] | 1,444,266 [10,109,861] | 2,901,554 [20,310,875] |
|  |  | Base | 6,893 [48,253] | 1,805,332 [12,637,327] | 3,626,942 [25,388,594] |
|  |  | High (+20%) | 8,272 [57,903] | 2,166,399 [15,164,792] | 4,352,330 [30,466,313] |
|  | 60–64 | Low (−20%) | 5,612 [39,282] | 1,650,991 [11,556,936] | 2,804,233 [19,629,632] |
|  |  | Base | 7,015 [49,102] | 2,063,738 [14,446,169] | 3,505,291 [24,537,040] |
|  |  | High (+20%) | 8,417 [58,922] | 2,476,486 [17,335,403] | 4,206,350 [29,444,448] |
|  | 65–69 | Low (−20%) | 4,892 [34,241] | 1,506,185 [10,543,295] | 2,411,232 [16,878,621] |
|  |  | Base | 6,115 [42,802] | 1,882,731 [13,179,118] | 3,014,039 [21,098,276] |
|  |  | High (+20%) | 7,337 [51,362] | 2,259,277 [15,814,942] | 3,616,847 [25,317,931] |
|  | 70–74 | Low (−20%) | 3,111 [21,776] | 1,045,833 [7,320,832] | 1,584,970 [11,094,792] |
|  |  | Base | 3,889 [27,220] | 1,307,291 [9,151,040] | 1,981,213 [13,868,490] |
|  |  | High (+20%) | 4,666 [32,664] | 1,568,750 [10,981,248] | 2,377,455 [16,642,188] |
|  | 75–79 | Low (−20%) | 2,260 [15,817] | 856,298 [5,994,087] | 1,215,906 [8,511,343] |
|  |  | Base | 2,824 [19,771] | 1,070,373 [7,492,609] | 1,519,883 [10,639,179] |
|  |  | High (+20%) | 3,389 [23,726] | 1,284,447 [8,991,131] | 1,823,859 [12,767,015] |
|  | 80+ | Low (−20%) | 2,550 [17,848] | 1,335,804 [9,350,627] | 2,053,352 [14,373,462] |
|  |  | Base | 3,187 [22,310] | 1,669,755 [11,688,284] | 2,566,690 [17,966,827] |
|  |  | High (+20%) | 3,825 [26,772] | 2,003,706 [14,025,941] | 3,080,028 [21,560,193] |
|  | Total | Low (−20%) | 29,015 [203,104] | 9,044,138 [63,308,964] | 15,538,041 [108,766,284] |
|  |  | Base | 36,269 [253,880] | 11,305,172 [79,136,205] | 19,422,551 [135,957,855] |
|  |  | High (+20%) | 43,522 [304,656] | 13,566,206 [94,963,445] | 23,307,061 [163,149,426] |
| RZV | 50–54 | Low (−20%) | 6,841 [47,888] | 1,623,444 [11,364,107] | 3,458,817 [24,211,716] |
|  |  | Base | 8,552 [59,861] | 2,029,305 [14,205,134] | 4,323,521 [30,264,644] |
|  |  | High (+20%) | 10,262 [71,833] | 2,435,166 [17,046,161] | 5,188,225 [36,317,573] |
|  | 55–59 | Low (−20%) | 7,431 [52,017] | 1,946,183 [13,623,280] | 3,909,913 [27,369,390] |
|  |  | Base | 9,289 [65,022] | 2,432,729 [17,029,100] | 4,887,391 [34,221,737] |
|  |  | High (+20%) | 11,147 [78,026] | 2,919,274 [20,434,920] | 5,864,869 [41,054,085] |
|  | 60–64 | Low (−20%) | 9,000 [63,000] | 2,647,883 [18,535,178] | 4,497,469 [31,482,283] |
|  |  | Base | 11,250 [78,750] | 3,309,853 [23,168,972] | 5,621,836 [39,352,854] |
|  |  | High (+20%) | 13,500 [94,500] | 3,971,824 [27,802,767] | 6,746,206 [47,223,425] |
|  | 65–69 | Low (−20%) | 7,845 [54,917] | 2,415,641 [16,909,486] | 3,867,167 [27,070,172] |
|  |  | Base | 9,807 [68,646] | 3,019,551 [21,136,857] | 4,833,959 [33,837,715] |
|  |  | High (+20%) | 11,768 [82,375] | 3,623,461 [25,364,229] | 5,800,751 [40,605,258] |
|  | 70–74 | Low (−20%) | 5,474 [38,321] | 1,840,397 [12,882,779] | 2,789,139 [19,523,976] |
|  |  | Base | 6,843 [47,901] | 2,300,496 [16,103,474] | 3,486,424 [24,404,970] |
|  |  | High (+20%) | 8,212 [57,481] | 2,760,596 [19,324,169] | 4,183,709 [29,285,964] |
|  | 75–79 | Low (−20%) | 3,976 [27,834] | 1,506,864 [10,548,050] | 2,139,682 [14,977,771] |
|  |  | Base | 4,970 [34,793] | 1,883,580 [13,185,062] | 2,674,602 [18,722,214] |
|  |  | High (+20%) | 5,964 [41,751] | 2,260,296 [15,822,075] | 3,209,522 [22,466,657] |
|  | 80+ | Low (−20%) | 4,776 [33,434] | 2,502,311 [17,516,180] | 3,846,467 [26,925,269] |
|  |  | Base | 5,970 [41,792] | 3,127,889 [21,895,225] | 4,808,084 [33,656,586] |
|  |  | High (+20%) | 7,164 [50,150] | 3,753,467 [26,274,271] | 5,769,700 [40,387,903] |
|  | Total | Low (−20%) | 45,344 [317,411] | 14,482,723 [101,379,060] | 24,508,654 [171,560,577] |
|  |  | Base | 56,681 [396,764] | 18,103,404 [126,723,825] | 30,635,817 [214,450,721] |
|  |  | High (+20%) | 68,017 [476,117] | 21,724,084 [152,068,590] | 36,762,981 [257,340,865] |
| HCS, healthcare system; HZ, herpes zoster; RZV, recombinant zoster vaccine; ZVL, live-attenuated vaccine. All cost estimates are reported in US dollars (1 USD = 1,300 Korean won). Values in [ ] represent estimates based on the assumed vaccination rate after reaching the target level following the introduction of national immunization program. | | | | | |

**Table C4.** Sensitivity analysis results for variations in the incidence rate of PHN

| Vaccine | Age, years | PHN incidence scenario | Expected number of PHN cases | Expected HCS cost savings from PHN cases, US dollars | Expected societal cost savings from PHN cases, US dollars |
| --- | --- | --- | --- | --- | --- |
| ZVL | 50–54 | Low (−20%) | 888 [6,214] | 1,098,324 [7,688,266] | 1,929,681 [13,507,769] |
|  |  | Base | 1,110 [7,767] | 1,372,905 [9,610,333] | 2,412,102 [16,884,711] |
|  |  | High (+20%) | 1,331 [9,320] | 1,647,486 [11,532,399] | 2,894,522 [20,261,653] |
|  | 55–59 | Low (−20%) | 1,155 [8,085] | 1,844,506 [12,911,541] | 3,442,372 [24,096,606] |
|  |  | Base | 1,444 [10,106] | 2,305,632 [16,139,426] | 4,302,965 [30,120,757] |
|  |  | High (+20%) | 1,733 [12,128] | 2,766,759 [19,367,311] | 5,153,558 [36,144,908] |
|  | 60–64 | Low (−20%) | 1,781 [12,465] | 5,392,575 [37,748,024] | 9,266,118 [64,862,826] |
|  |  | Base | 2,226 [15,582] | 6,740,719 [47,185,030] | 11,582,647 [81,078,532] |
|  |  | High (+20%) | 2,671 [18,698] | 8,088,862 [56,622,036] | 13,899,177 [97,294,239] |
|  | 65–69 | Low (−20%) | 2,063 [14,444] | 3,416,317 [23,914,222] | 5,413,967 [37,897,766] |
|  |  | Base | 2,579 [18,056] | 4,270,397 [29,892,777] | 6,767,458 [47,372,208] |
|  |  | High (+20%) | 3,095 [21,667] | 5,124,476 [35,871,333] | 8,120,950 [56,846,649] |
|  | 70–74 | Low (−20%) | 2,011 [14,076] | 4,279,688 [29,957,814] | 6,013,497 [42,094,481] |
|  |  | Base | 2,514 [17,596] | 5,349,610 [37,447,267] | 7,516,872 [52,618,102] |
|  |  | High (+20%) | 3,016 [21,115] | 6,419,532 [44,936,721] | 9,020,246 [63,141,722] |
|  | 75–79 | Low (−20%) | 1,886 [13,199] | 5,343,578 [37,405,046] | 7,353,289 [51,473,021] |
|  |  | Base | 2,357 [16,499] | 6679473 [46,756,308] | 9,191,611 [64,341,276] |
|  |  | High (+20%) | 2,828 [19,799] | 8,015,367 [56,107,569] | 11,029,933 [77,209,531] |
|  | 80+ | Low (−20%) | 3,007 [21,049] | 8,463,684 [59,245,788] | 13,824,825 [96,773,776] |
|  |  | Base | 3,759 [26,311] | 10579605 [74,057,235] | 17,281,032 [120,967,221] |
|  |  | High (+20%) | 4,511 [31,574] | 12,695,526 [88,868,682] | 20,737,238 [145,160,665] |
|  | Total | Low (−20%) | 12,790 [89,533] | 29,838,672 [208,870,701] | 47,243,749 [330,706,245] |
|  |  | Base | 15,988 [111,917] | 37,298,339 [261,088,376] | 59,054,687 [413,382,806] |
|  |  | High (+20%) | 19,186 [134,300] | 44,758,007 [313,306,051] | 70,865,624 [496,059,367] |
| RZV | 50–54 | Low (−20%) | 1,150 [8,050] | 1,422,866 [9,960,064] | 2,499,881 [17,499,166] |
|  |  | Base | 1,437 [10,062] | 1,778,583 [12,450,080] | 3,124,851 [21,873,957] |
|  |  | High (+20%) | 1,725 [12,074] | 2,134,299 [14,940,096] | 3,749,821 [26,248,749] |
|  | 55–59 | Low (−20%) | 1,496 [10,474] | 2,389,537 [16,726,759] | 4,459,555 [31,216,887] |
|  |  | Base | 1,870 [13,093] | 2,986,921 [20,908,448] | 5,574,444 [39,021,109] |
|  |  | High (+20%) | 2,244 [15,711] | 3,584,305 [25,090,138] | 6,689,333 [46,825,331] |
|  | 60–64 | Low (−20%) | 2,307 [16,149] | 6,986,021 [48,902,150] | 12,004,154 [84,029,077] |
|  |  | Base | 2,884 [20,186] | 8,732,527 [61,127,687] | 15,005,192 [105,036,346] |
|  |  | High (+20%) | 3,460 [24,223] | 10,479,032 [73,353,224] | 18,006,231 [126,043,615] |
|  | 65–69 | Low (−20%) | 2,673 [18,713] | 4,425,802 [30,980,611] | 7,013,734 [49,096,139] |
|  |  | Base | 3,342 [23,391] | 5,532,252 [38,725,764] | 8,767,168 [61,370,173] |
|  |  | High (+20%) | 4,010 [28,069] | 6,638,702 [46,470,917] | 10,520,601 [73,644,208] |
|  | 70–74 | Low (−20%) | 2,605 [18,236] | 5,544,288 [38,810,018] | 7,790,420 [54,532,937] |
|  |  | Base | 3,256 [22,795] | 6,930,360 [48,512,522] | 9,738,024 [68,166,171] |
|  |  | High (+20%) | 3,908 [27,354] | 8,316,432 [58,215,027] | 11,685,629 [81,799,406] |
|  | 75–79 | Low (−20%) | 2,443 [17,100] | 6,922,546 [48,457,825] | 9,526,104 [66,682,731] |
|  |  | Base | 3,053 [21,374] | 8,653,183 [60,572,282] | 11,907,631 [83,353,414] |
|  |  | High (+20%) | 3,664 [25,649] | 10,383,820 [72,686,738] | 14,289,157 [100,024,097] |
|  | 80+ | Low (−20%) | 3,896 [27,269] | 10,964,609 [76,752,266] | 17,909,909 [125,369,362] |
|  |  | Base | 4,869 [34,086] | 13,705,762 [95,940,333] | 22,387,386 [156,711,702] |
|  |  | High (+20%) | 5,843 [40,903] | 16,446,914 [115,128,399] | 26,864,863 [188,054,043] |
|  | Total | Low (−20%) | 16,570 [115,989] | 38,655,670 [270,589,693] | 61,203,757 [428,426,299] |
|  |  | Base | 20,712 [144,987] | 48,319,588 [338,237,116] | 76,504,696 [535,532,873] |
|  |  | High (+20%) | 24,855 [173,984] | 57,983,506 [405,884,540] | 91,805,635 [642,639,448] |
| HCS, healthcare system; PHN, postherpetic neuralgia; RZV, recombinant zoster vaccine; ZVL, live-attenuated vaccine. All cost estimates are reported in US dollars (1 USD = 1,300 Korean won). Values in [ ] represent estimates based on the assumed vaccination rate after reaching the target level following the introduction of national immunization program. | | | | | |

**Table C5.** Scenario analysis of RZV two-dose completion (assuming 70% dose-1 uptake) and resulting effective full-series coverage.

| RZV second-dose completion rate among dose-1 recipients^a^, % | Age, years | Expected number of HZ cases prevented by RZV (two doses) | Expected number of PHN cases prevented by RZV (two doses) | Expected HCS cost savings from HZ+PHN cases prevented by RZV (two doses), US dollars | Expected societal cost savings from HZ+PHN cases prevented by RZV (two doses), US dollars |
| --- | --- | --- | --- | --- | --- |
| 50.00% | 50–54 | 29,930 | 5,031 | 13,327,607 | 26,069,301 |
|  | 55–59 | 32,511 | 6,546 | 18,968,774 | 36,616,423 |
|  | 60–64 | 39,375 | 10,093 | 42,148,330 | 72,194,600 |
|  | 65–69 | 34,323 | 11,695 | 29,931,311 | 47,603,944 |
|  | 70–74 | 23,950 | 11,397 | 32,307,998 | 46,285,571 |
|  | 75–79 | 17,396 | 10,687 | 36,878,672 | 51,037,814 |
|  | 80+ | 20,896 | 17,043 | 58,917,779 | 95,184,144 |
|  | Total | 198,382 | 72,493 | 232,480,471 | 374,991,797 |
| 60.00% | 50–54 | 35,916 | 6,037 | 15,993,128 | 31,283,161 |
|  | 55–59 | 39,013 | 7,856 | 22,762,529 | 43,939,708 |
|  | 60–64 | 47,250 | 12,112 | 50,577,996 | 86,633,520 |
|  | 65–69 | 41,188 | 14,034 | 35,917,573 | 57,124,733 |
|  | 70–74 | 28,740 | 13,677 | 38,769,598 | 55,542,685 |
|  | 75–79 | 20,876 | 12,825 | 44,254,406 | 61,245,377 |
|  | 80+ | 25,075 | 20,452 | 70,701,335 | 114,220,973 |
|  | Total | 238,058 | 86,992 | 278,976,565 | 449,990,156 |
| 70.00% | 50–54 | 41,902 | 7,043 | 18,658,650 | 36,497,021 |
|  | 55–59 | 45,515 | 9,165 | 26,556,284 | 51,262,992 |
|  | 60–64 | 55,125 | 14,130 | 59,007,661 | 101,072,440 |
|  | 65–69 | 48,052 | 16,374 | 41,903,835 | 66,645,522 |
|  | 70–74 | 33,530 | 15,956 | 45,231,197 | 64,799,799 |
|  | 75–79 | 24,355 | 14,962 | 51,630,141 | 71,452,940 |
|  | 80+ | 29,254 | 23,860 | 82,484,891 | 133,257,802 |
|  | Total | 277,735 | 101,491 | 325,472,659 | 524,988,516 |
| 80.00% | 50–54 | 47,888 | 8,050 | 21,324,171 | 41,710,881 |
|  | 55–59 | 52,017 | 10,474 | 30,350,039 | 58,586,277 |
|  | 60–64 | 63,000 | 16,149 | 67,437,327 | 115,511,360 |
|  | 65–69 | 54,917 | 18,713 | 47,890,097 | 76,166,311 |
|  | 70–74 | 38,321 | 18,236 | 51,692,797 | 74,056,913 |
|  | 75–79 | 27,834 | 17,100 | 59,005,875 | 81,660,503 |
|  | 80+ | 33,434 | 27,269 | 94,268,447 | 152,294,631 |
|  | Total | 317,411 | 115,989 | 371,968,753 | 599,986,875 |
| 90.00% | 50–54 | 53,874 | 9,056 | 23,989,693 | 46,924,741 |
|  | 55–59 | 58,519 | 11,783 | 34,143,793 | 65,909,561 |
|  | 60–64 | 70,875 | 18,167 | 75,866,993 | 129,950,280 |
|  | 65–69 | 61,782 | 21,052 | 53,876,359 | 85,687,099 |
|  | 70–74 | 43,111 | 20,515 | 58,154,397 | 83,314,027 |
|  | 75–79 | 31,313 | 19,237 | 66,381,610 | 91,868,066 |
|  | 80+ | 37,613 | 30,677 | 106,052,002 | 171,331,460 |
|  | Total | 357,087 | 130,488 | 418,464,847 | 674,985,235 |
| 100.00% | 50–54 | 59,861 | 10,062 | 26,655,214 | 52,138,602 |
|  | 55–59 | 65,022 | 13,093 | 37,937,548 | 73,232,846 |
|  | 60–64 | 78,750 | 20,186 | 84,296,659 | 144,389,200 |
|  | 65–69 | 68,646 | 23,391 | 59,862,621 | 95,207,888 |
|  | 70–74 | 47,901 | 22,795 | 64,615,996 | 92,571,141 |
|  | 75–79 | 34,793 | 21,374 | 73,757,344 | 102,075,628 |
|  | 80+ | 41,792 | 34,086 | 117,835,558 | 190,368,288 |
|  | Total | 396,764 | 144,987 | 464,960,941 | 749,983,594 |
| HCS, healthcare system; HZ, herpes zoster; PHN, postherpetic neuralgia; RZV, recombinant zoster vaccine; ZVL, live-attenuated vaccine. All cost estimates are reported in US dollars (1 USD = 1,300 Korean won).  ^a^Dose-1 uptake was fixed at 70% under the NIP assumption. “RZV 2-dose completion rate” refers to the proportion of dose-1 recipients who received dose 2. Therefore, the implied effective full-series (two-dose series) coverage equals 0.70 × completion rate, ranging from 35% (50% completion) to 70% (100% completion). Because robust and generalizable effectiveness estimates for a single RZV dose were not available for base-case parameterization, we conservatively assumed zero protection for incomplete regimens (one dose only) in this scenario analysis. | | | | | |
